# Biosynthesis and Immunological Evaluation of a Dual-Antigen Nanoconjugate Vaccine Targeting Group A Streptococcus

**DOI:** 10.3390/vaccines14030237

**Published:** 2026-03-04

**Authors:** Xiaoxia Li, Xiang Wang, Decong Kong, Hua Jiang, Ying Chen, Wenhua Huang, Yongqiang Jiang

**Affiliations:** 1 State Key Laboratory of Pathogen and Biosecurity, Academy of Military Medical Sciences, Beijing 100071, China; xiaoxialimail@126.com (X.L.); wangnit1999@163.com (X.W.); kongdecong-118@163.com (D.K.); jhua76@126.com (H.J.); 2 School of Light Industry Science and Engineering, Beijing Technology and Business University, Beijing 100048, China; chenying@btbu.edu.cn

**Keywords:** Group A Streptococcus, PGCT, glycoconjugates, nanoparticle, biosynthesis

## Abstract

**Background**: Group A Streptococcus (GAS) induces a wide spectrum of human diseases, ranging from superficial infections to life-threatening invasive conditions and post-infectious sequelae such as rheumatic heart disease, posing a heavy global health burden. Critically, there is still no licensed commercial vaccine against GAS, making the development of novel, effective vaccines against this pathogen an urgent and crucial unmet medical need. **Methods**: We developed a dual-antigen nanoconjugate vaccine against GAS. The Group A Carbohydrate polyrhamnose backbone (GAC^PR^) and truncated SLO were site-specifically conjugated via Protein Glycan Coupling Technology (PGCT) in engineered *E. coli*, and then linked to ferritin nanoparticles using the SnoopTag/SnoopCatcher system. Safety, immunogenicity, and protective efficacy were evaluated in murine models. **Results**: The nanovaccine was successfully synthesized with high purity. It elicited robust GAC- and SLO-specific IgG/IgG1 responses, conferred 90% survival against lethal GAS challenge (vs. 0–50% in controls), reduced bacterial loads in organs, and lowered inflammatory cytokines. Passive immunization with vaccine-induced serum also achieved 90% survival. No abnormal biochemical indicators, inflammatory responses, or organ pathology were observed. **Conclusions**: This study successfully developed a bivalent nanoparticle vaccine against GAS. This novel nanovaccine exhibits excellent safety, strong immunogenicity, and effective protection against GAS, providing a promising vaccine candidate.

## 1. Introduction

Group A Streptococcus (GAS), also known as *Streptococcus pyogenes*, is a major Gram-positive bacterial pathogen responsible for a broad spectrum of human diseases [1,2]. Superficial infections typically present as acute pharyngitis, tonsillitis, or impetigo, whereas invasive infections can progress to life-threatening conditions such as necrotizing fasciitis and streptococcal toxic shock syndrome (STSS) [3]. In addition, inadequately treated GAS infections may lead to post-infectious immune-mediated sequelae, including acute glomerulonephritis and acute rheumatic fever (ARF) [4]. If unresolved, ARF can further develop into rheumatic heart disease (RHD) [5], which remains a leading cause of GAS-associated mortality worldwide.

GAS poses a substantial global public health burden, particularly in developing countries, with an estimated 800 million infections and approximately 639,000 deaths each year [6]. The outbreak in the United Kingdom in late 2022, resulting in 772 reported infections and 61 deaths, further highlighted the urgent need for effective prevention and control strategies [7]. Despite decades of intensive research, no licensed vaccine against GAS is currently available [8].

The Group A Carbohydrate (GAC), a major and highly conserved component of the GAS cell wall, represents a promising vaccine candidate [1,9]. Structurally, GAC is composed of a polyrhamnose backbone decorated with N-acetylglucosamine (GlcNAc) side chains [10]. However, the GlcNAc moiety has been implicated in triggering autoimmune responses associated with rheumatic heart disease (RHD) [11,12]. To address this safety concern, a GlcNAc-deficient variant, known as the GAC polyrhamnose backbone (GAC^PR^), was developed [13]. GAC^PR^ preserves the immunogenic polyrhamnose scaffold while eliminating the cross-reactive GlcNAc epitope. Previous studies have shown that GAC^PR^-based conjugate vaccines elicit robust opsonic antibody responses and provide protective immunity in animal models, without inducing autoimmune reactivity [14,15].

Traditional approaches for producing GAC-based vaccines rely on chemical extraction or total chemical synthesis [16], both of which face significant limitations, including biosafety concerns, potential contamination with bacterial proteins (such as M protein), and difficulties in faithfully reproducing the native polysaccharide conformation. Protein Glycan Coupling Technology (PGCT) has emerged as an innovative and highly efficient strategy for the biosynthesis of bacterial glycoconjugate vaccines [17,18]. This method relies on the in vivo coupling of recombinant glycan antigens to an immunogenic carrier protein within engineered bacterial cells, offering a highly customizable platform for rational vaccine design. At the core of this technology are bacterial oligosaccharyltransferases (OSTs), a class of enzymes that catalyze the glycosylation of substrate proteins. To date, four OSTs have been established as viable tools for producing polysaccharide conjugate vaccines: the N-oligosaccharyltransferase PglB, and the O-oligosaccharyltransferases PglL [19], PglS [20], and TfpM [21]. PglL exhibits exceptionally broad substrate specificity, enabling the transfer of glycans that contain either a galactose residue at the reducing end or a sugar bearing an acetamido group at the C2 position [19]. Leveraging the PGCT system, researchers have successfully developed glycoconjugate vaccines against various pathogens, including *Shigella dysenteriae*, *Shigella flexneri*, extraintestinal pathogenic *Escherichia coli* (ExPEC), and *Francisella tularensis* [22,23,24]. Notably, a tetravalent ExPEC vaccine synthesized via PGCT has advanced to clinical trials, underscoring the strong translational potential of this technology.

To further enhance the immunogenicity of T cell-independent polysaccharide antigens, nanoparticle-based delivery systems have emerged as a powerful strategy [25]. Nanocarriers, including virus-like particles (VLPs) and ferritin, enhance antigen presentation, promote efficient lymphatic trafficking, and facilitate robust activation of both B-cell and T-cell responses while maintaining favorable safety profiles [26,27,28]. Moreover, the incorporation of protein antigens offers the potential to broaden protective coverage and improve overall vaccine efficacy.

Here, we report the development of a novel dual-antigen nanoconjugate vaccine against GAS. We employed PGCT for site-directed conjugation between the GAC^PR^ and truncated SLO protein in engineered *E*. *coli*. The resulting glycoprotein was subsequently displayed on ferritin nanoparticles through a SnoopTag/SnoopCatcher (SnT/SnC) conjugation system. This modular design enables the co-delivery of both polysaccharide and protein antigens within a single nanoplatform. Our data demonstrate that this nanovaccine exhibits an excellent safety profile and elicits robust humoral immune responses, conferring significant protection against GAS challenge in a murine model. Collectively, this work not only identifies a promising vaccine candidate against GAS but also establishes a versatile and expandable platform for the rational co-delivery of diverse antigens.

## 2. Materials and Methods

### 2.1. Strains and Animals

All *E. coli* strains were cultured in Luria–Bertani broth (BD Biosciences, San Jose, CA, USA). Solid media contained 1.5% agar. For plasmid selection, the antibiotics were used at the following concentrations: ampicillin (100 μg/mL), and kanamycin (50 μg/mL). GAS strain MGAS5005 was obtained from ATCC (BAA-947) and corresponded to the serotype M1. GAS was grown in Todd Hewitt Yeast (Todd Hewitt broth (BD Biosciences, San Jose, CA, USA) plus 2‰ yeast extract) at 37 °C. Pathogen-free female ICR mice (6–8 weeks old) were purchased from SPF Biotechnology (Beijing, China) and housed at the Laboratory Animal Centre of the Academy of Military Medical Sciences. All the animal experiments were approved by the Academy of Military Medical Sciences Animal Care and Use Committee (Ethics Approval Code IACUC-DWZX-2024-P007).

### 2.2. Construction of Polysaccharide-Expressing Plasmid

The *GacA-G* gene cluster responsible for GAC^PR^ biosynthesis was amplified from the genomic DNA of *Streptococcus pyogenes* strain MGAS5005. The pBBR1MCS-2 plasmid backbone was linearized by PCR amplification. The amplified gene cluster and linearized vector were assembled by homologous recombination using the pEASY-Basic Seamless Cloning and Assembly Kit (TransGen Biotech, Beijing, China). The assembly reaction was incubated at 50 °C for 40 min, followed by chemical transformation into *E. coli* NEB 10-beta competent cells. Transformants were selected on LB agar plates containing kanamycin and incubated at 37 °C overnight. Positive clones were screened by colony PCR. Plasmids were subsequently isolated from confirmed clones, and the integrity of the inserted sequence was verified by KBSeq (Sangon Biotech, Shanghai, China).

### 2.3. Construction of Protein-Expressing Plasmid

The gene encoding the Tac promoter-DsbA signal peptide-SLO(ΔC101)-SnoopCatcher-glycosylation modification sequence 4573 was chemically synthesized by Sangon Biotech (Shanghai, China). The synthesized fragment was inserted into the pET-PglL-CTB^4573C^ vector [19] via restriction enzyme digestion and ligation, replacing the CTB gene to generate the recombinant plasmid pET28a-PglL-SLO(ΔC101)-SnC^4573C^. This construct was designed to co-express the oligosaccharyltransferase PglL and the fusion substrate protein SLO(ΔC101)-SnC^4573^. To ensure compatibility with the polysaccharide expression plasmid in the *E. coli* chassis strain, the DNA fragments encoding PglL and the substrate protein were further subcloned into the pET32a vector, resulting in the final plasmid pET32a-PglL-SLO(ΔC101)-SnC^4573C^. This recombinant plasmid was subsequently used for glycoprotein production experiments.

### 2.4. Glycoprotein Expression and Purification in E. coli

*E. coli* W3110 Δ*waaL* Δ*wbbH-L* cells [29] were rendered electrocompetent by cultivation to the mid-logarithmic phase, followed by four successive washes with 10% (*v*/*v*) glycerol and a final resuspension in 1/200 of the original culture volume. The cells were first electroporated with GAC^PR^-expressing plasmids and selected on LB agar plates supplemented with kanamycin. Individual colonies were then picked, rendered electrocompetent as described above, and subjected to a second round of electroporation with pET32a-PglL-SLO(ΔC101)-SnC^4573C^. Transformants were selected on LB agar supplemented with kanamycin and ampicillin. Selected colonies were used to inoculate 250 mL starter cultures in LB medium containing the appropriate antibiotics and grown overnight at 37 °C with shaking at 180 rpm. The starter cultures were subsequently used to inoculate multiple 2 L Erlenmeyer flasks, each containing 1 L of LB medium supplemented with antibiotics and 0.5 mM IPTG. Cultures were grown for 16 h at 28 °C with shaking at 180 rpm.

Cells were harvested by centrifugation and resuspended in buffer A (20 mM phosphate buffer, 500 mM NaCl, 20 mM imidazole, pH 8.0). Cell lysis was performed using a homogenizer, and the lysate was clarified by centrifugation. The resulting supernatant was loaded onto a HisTrap HP affinity column (Cityva, Washington, DC, USA). After washing with buffer A, bound proteins were eluted with buffer B (20 mM phosphate buffer, 500 mM NaCl, 500 mM imidazole, pH 8.0). Eluted fractions containing glycoproteins were confirmed by SDS–PAGE and subsequently concentrated using ultrafiltration. The concentrated sample was further purified by size-exclusion chromatography on a Superdex 200 Prep Grade column using PBS as the mobile phase at a flow rate of 1 mL/min. Protein-containing fractions were collected sequentially and analyzed by Coomassie Brilliant Blue staining. For the subsequent nanoparticle conjugation steps, we specifically pooled and utilized only the high-molecular-weight fractions corresponding to the fully glycosylated protein to ensure the purity of our starting material.

### 2.5. Preparation of Ferritin Nanoparticle

*Pyrococcus furiosus* ferritin [30] was chemically synthesized by GenScript (Nanjing, China). A SnoopTag was fused to ferritin via a flexible (GGGGS)_3_ linker, and the resulting fusion gene was cloned into the pET28a vector to express SnT–ferritin nanoparticles in *E. coli* BL21(DE3). Protein expression was induced with 100 μM IPTG and carried out overnight at 18 °C. Bacterial cells were harvested by centrifugation and resuspended in Buffer A (20 mM Tris–HCl, pH 8.0). Cells were disrupted using a homogenizer, and the lysate was clarified by centrifugation. The clarified supernatant was heat-treated at 70 °C for 15 min to precipitate heat-labile proteins, followed by a second centrifugation step to recover the heat-stable fraction. After a 20-fold dilution with Buffer A, the recovered supernatant was loaded onto a HiTrap Q HP anion-exchange column (Cytiva, Washington, DC, USA). After extensive washing with Buffer A, bound proteins were eluted using a continuous linear gradient of Buffer B (20 mM Tris–HCl, 500 mM NaCl, pH 8.0), reaching 50% Buffer B over 50 min. The eluted SnT–ferritin nanoparticles were concentrated using an Amicon ultrafiltration device (Millipore, Burlington, MA, USA) (100 kDa molecular weight cutoff) and further purified by size-exclusion chromatography (SEC) on a Superdex 200 Prep Grade column equilibrated with PBS.

### 2.6. Preparation of Nanovaccines

The purified SnC-fused protein was incubated with SnT–Fer at different molar ratios overnight at 4 °C, and the formation of conjugated products was assessed by SDS–PAGE. In this study, SnT–Fer was incubated with a twofold molar excess of GAC^PR^-modified SnC–SLO(ΔC101) (GAC^PR^–SLO(ΔC101)) to generate the nanoconjugate GAC^PR^–SLO(ΔC101)–Fer. Following the conjugation reaction, the mixture was purified by SEC using a Superdex 200 Prep Grade column. Fractions containing the target conjugate were collected and analyzed by SDS–PAGE. The hydrodynamic size distribution of the resulting nanoparticles was characterized by dynamic light scattering (DLS) using a Zetasizer Ultra instrument (Malvern Panalytical, Malvern, UK).

### 2.7. Western Blot Analysis

Overnight cultures of *E. coli* W3110 harboring the GAC^PR^ expression plasmid were collected by centrifugation from 1 mL aliquots. Cell pellets were resuspended in 75 μL PBS, mixed with 25 μL 6 × SDS loading buffer, and boiled for 15 min. Proteinase K (2 μL) was subsequently added, and the samples were digested at 56 °C for 1 h. After centrifugation, the supernatants were collected and subjected to SDS–PAGE analysis. Either 8% homogeneous or 4–20% gradient gels were utilized depending on specific experimental requirements. All precast protein gels and the corresponding MOPS running buffer were purchased from GenScript (Nanjing, China). Electrophoresis was performed at a constant voltage of 180 V for 50 min. Proteins were transferred onto nitrocellulose membranes, which were then blocked with blocking buffer (TBS containing 5% BSA). GAC expression was detected using a rabbit anti-GAC antibody (Abcam, Cambridge, UK), followed by incubation with an IRDye 800CW goat anti-rabbit IgG secondary antibody (LI-COR biosciences, Lincoln, NE, USA). Membranes were visualized using a LI-COR Odyssey XF imaging system (LI-COR Biosciences, Lincoln, NE, USA).

For glycoprotein expression analysis, *E. coli* W3110Δ*waaL*Δ*wbbH-L* co-harboring the polysaccharide biosynthesis plasmid and the carrier protein expression plasmid was grown overnight. A 1 mL aliquot of the culture was pelleted by centrifugation, resuspended in 75 μL PBS, and mixed with 25 μL 6 × SDS loading buffer. Following boiling for 15 min and centrifugation, the supernatant was analyzed by SDS–PAGE. GAC^PR^ and SLO(ΔC101)-SnC were detected using a rabbit anti-GAC antibody and a mouse anti-His tag antibody (Abmart, Shanghai, China), respectively. IRDye 680RD goat anti-mouse IgG secondary antibody (LI-COR biosciences, Lincoln, NE, USA) and IRDye 800CW goat anti-rabbit IgG secondary antibody (LI-COR biosciences, Lincoln, NE, USA) were used for signal detection.

### 2.8. Anthrone Assay for Total Polysaccharide Concentration

A rhamnose stock solution was prepared at a concentration of 200 μg/mL. Standard solutions with final concentrations of 0, 10, 20, 40, 60, 80, and 100 μg/mL were generated by serial dilution of the stock solution. Subsequently, 250 μL aliquots of each standard solution and glycoprotein sample were transferred into test tubes. Anthrone reagent was prepared at a concentration of 2 mg/mL (Sigma-Aldrich, St. Louis, MO, USA) in ice-cold sulfuric acid, and 1 mL of this reagent was added to each tube. The mixtures were immediately vortexed, placed on ice, and then incubated in a boiling water bath for 15 min. After incubation, the tubes were rapidly cooled to room temperature on ice. Finally, 200 μL aliquots from each reaction mixture were transferred to a microplate, and the absorbance was measured at 620 nm using a microplate reader SpectraMax i3X (Molecular Devices, San Jose, CA, USA). A standard curve was generated based on the rhamnose standards and used to determine the carbohydrate content of the glycoprotein samples.

### 2.9. Negative Staining Electron Microscopy

Nanoparticles were subjected to negative-stain transmission electron microscopy (TEM) analysis. Briefly, a 3 μL aliquot of the sample was applied to a 230-mesh copper grid coated with a carbon film that had been rendered hydrophilic by glow discharge. After incubation for 60 s, excess liquid was carefully blotted from the edge of the grid using filter paper, taking care to avoid drying of the grid. The grid was subsequently washed twice with 3 μL of 2% (*w*/*v*) uranyl acetate solution and then stained with 3 μL of the same solution for 60 s. Excess stain was removed by blotting with filter paper, and the grid was allowed to air-dry. Imaging was performed using a transmission electron microscope (Hitachi, Tokyo, Japan)

### 2.10. Extraction of LPS

*E. coli* W3110 harboring the GAC^PR^ expression plasmid was harvested by centrifugation and resuspended in distilled water (ddH_2_O). An equal volume of 90% phenol was added, and the suspension was vigorously mixed and incubated at 68 °C for 30 min. The mixture was then centrifuged at 8000 rpm for 20 min at 4 °C, and the aqueous phase was carefully collected. Residual phenol was removed by dialysis against ddH_2_O for 48 h with multiple water changes. The dialyzed sample was subsequently treated sequentially with DNase (5 μg/mL), RNase (1 μg/mL), and proteinase K (20 μg/mL), with incubation at the respective optimal temperatures for each enzyme. After enzymatic digestion, the solution was boiled for 10 min to inactivate the enzymes and centrifuged at 8000 rpm for 10 min. The resulting supernatant containing lipopolysaccharides (LPS) was collected for further analysis.

### 2.11. Animal Immunization Experiments

Specific-pathogen-free (SPF) female ICR mice were purchased from SPF Biotechnology (Beijing, China) and housed at the Laboratory Animal Center of the Academy of Military Medical Sciences. Six-week-old mice were randomly assigned into four groups and immunized three times at two-week intervals with 100 μL of PBS, ferritin (Fer), GAC^PR^-SLO(ΔC101), or GAC^PR^-SLO(ΔC101)-Fer, respectively. All immunizations were administered subcutaneously and contained 2.5 μg of GAC polysaccharide. Mice in the Fer group received an amount of ferritin matched to the protein content of the GAC^PR^-SLO(ΔC101)-Fer conjugate.

Tail vein blood samples were collected seven days after each immunization. Serum was separated by centrifugation and stored at −80 °C until further analysis. Two weeks after the final immunization, mice were challenged intraperitoneally with 5 × 10^8^ colony-forming units (CFU) of *Streptococcus pyogenes* strain MGAS5005, and survival was monitored daily. For the low-dose infection model, mice were intraperitoneally infected with 1 × 10^8^ CFU of MGAS5005. Body weights were recorded daily following infection. Blood samples were collected from the tail vein at 1 day post-infection, and serum levels of tumor necrosis factor-α (TNF-α) were quantified using a mouse TNF-α ELISA kit (ExCell Bio, Suzhou, China).

At 3 days post-infection, mice were euthanized, and liver and spleen tissues were aseptically harvested. The tissues were homogenized in sterile PBS, serially diluted, and plated on agar plates. Bacterial burdens in the organs were determined by enumerating colony-forming units.

### 2.12. ELISA

To exclude potential interference from the His tag in the immunization results, purified glutathione S-transferase-tagged SLO(ΔC101) (GST-SLO(ΔC101)) was used as the coating antigen for enzyme-linked immunosorbent assay (ELISA). Briefly, GST-SLO(ΔC101) (0.1 μg per well) or GAC^PR^ LPS (5 μg per well) was precoated onto 96-well immunoplates at 4 °C overnight. The plates were washed three times with phosphate-buffered saline containing 0.05% Tween 20 (PBST), patted dry, and blocked with blocking buffer (PBST supplemented with 5% bovine serum albumin) at 4 °C overnight. After blocking, the plates were washed and incubated with serially diluted mouse sera at 37 °C for 1 h. The plates were then washed three times with PBST and incubated with horseradish peroxidase (HRP)-conjugated goat anti-mouse IgG or IgG1 secondary antibodies (1:50,000 dilution; Abcam, Cambridge, UK) at 37 °C for 1 h. Following another washing step, color development was initiated using a TMB Single-Component Substrate Solution (Solarbio, Beijing, China) and stopped with stop solution. Absorbance was measured at 450 nm using a microplate reader.

### 2.13. Safety Determination

Six-week-old specific-pathogen-free female ICR mice were randomly divided into two groups (*n* = 5 per group). One group was immunized with GAC^PR^-SLO(ΔC101)-Fer (equivalent to 2.5 μg GAC^PR^), while the control group received phosphate-buffered saline (PBS). Blood samples were collected from the tail vein at 1, 3, and 7 days after the final immunization. Serum was separated by centrifugation, and the concentrations of proinflammatory cytokines, including tumor necrosis factor-α (TNF-α), interferon-γ (IFN-γ), and interleukin-6 (IL-6), were quantified using commercial mouse ELISA kits (ExCell bio, Suzhou, China), according to the manufacturer’s instructions.

On day 7 post-immunization, additional blood samples were collected from the tail vein to evaluate serum biochemical indicators of organ function. Levels of alanine aminotransferase (ALT) and aspartate aminotransferase (AST) (markers of liver function), blood urea nitrogen (BUN) (a marker of renal function) and lactate dehydrogenase (LDH) (an indicator of general tissue damage) were measured using a Chemray 240 automatic biochemical analyzer (Rayto, Shenzhen, China). Subsequently, mice were euthanized, and major organs, including the heart, liver, spleen, lungs, and kidneys, were harvested and fixed in 4% paraformaldehyde (Solarbio, Beijing, China) for histopathological examination.

### 2.14. Hematoxylin and Eosin (H&E) Staining

Hematoxylin and eosin (H&E) staining was performed by Wuhan Service Biotechnology Co., Ltd. (Wuhan, China), including tissue collection, fixation, embedding, and preparation of both paraffin-embedded and frozen sections. Paraffin sections were sequentially deparaffinized, rehydrated through graded ethanol solutions (100% and 75%), and rinsed with distilled water. Frozen sections were equilibrated to room temperature and fixed using an appropriate tissue fixative. Subsequently, all sections were treated with an HD constant staining pretreatment solution, followed by hematoxylin staining. After rinsing, sections were subjected to differentiation and bluing steps, then counterstained with eosin. Finally, the sections were dehydrated, cleared with xylene, and mounted with a neutral resin mounting medium. Histopathological examination and image acquisition were performed using light microscopy (Olympus, Tokyo, Japan).

### 2.15. Statistical Analysis

Antibody titers and bacterial loads were lg-transformed. Statistical analyses were conducted using GraphPad Prism version 8.0 (GraphPad Software, San Diego, CA, USA). Data were analyzed using the Kruskal–Wallis test or one-way analysis of variance (ANOVA) with Dunn’s multiple comparison test. All the data were expressed as means ± standard deviations (SD). Values of *p* < 0.05 were considered statistically significant (**** *p* < 0.0001, *** *p* < 0.001, ** *p* < 0.01, and * *p* < 0.05)

## 3. Results

### 3.1. Design and Production of a Dual-Antigen Nanoconjugate Vaccine Against GAS

GAC^PR^ is regarded as a promising vaccine candidate due to its high degree of conservation across GAS strains. However, as a T cell–independent antigen, polysaccharides alone are inefficient at inducing long-lasting immune memory and therefore require conjugation to a carrier protein enriched in T cell epitopes. In addition, the immunoprotective efficacy of GAC^PR^ polysaccharide as a standalone antigen against GAS remains suboptimal, underscoring the necessity of combining it with highly immunogenic endogenous GAS proteins.

**Figure 1:**
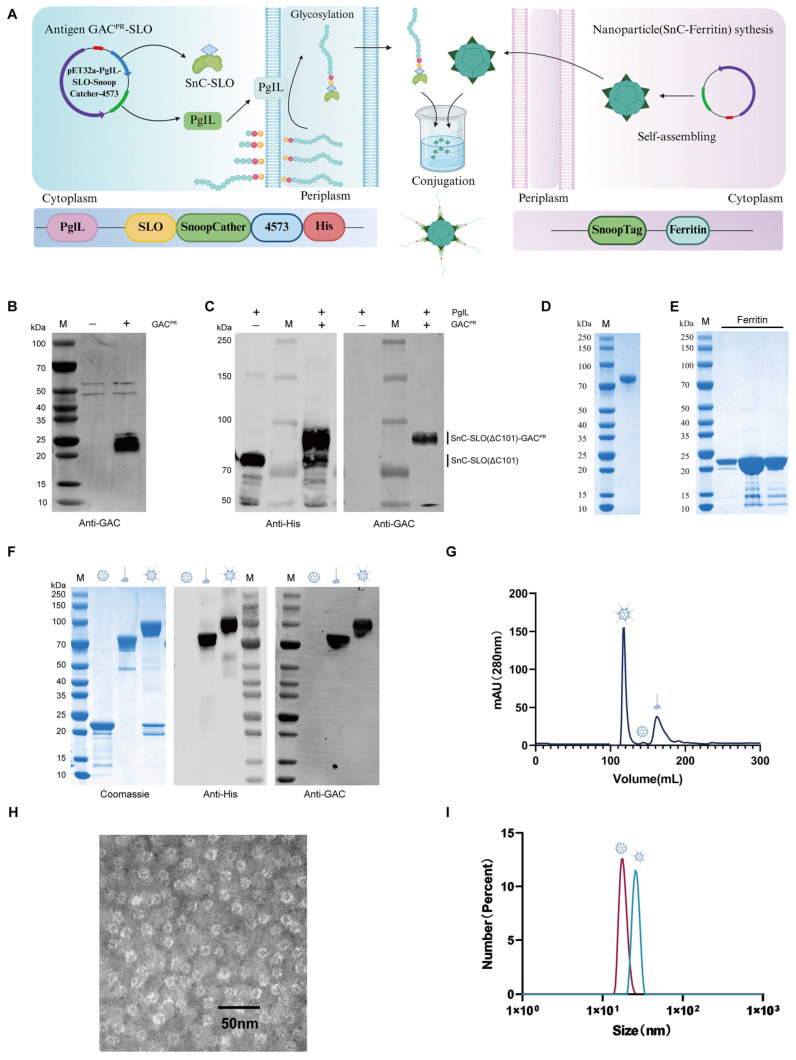
Production and characterization of the nanoscale conjugate vaccine. (**A**) Schematic illustration of nanovaccine preparation. Created with BioRender.com. (**B**) Western blot analysis of GAC^PR^ expression using anti-GAC commercial antibody. (**C**) Western blot analysis of glycoprotein SnC-SLO (ΔC101)-GAC^PR^ expression. (**D**) SDS-PAGE analysis of purified glycoprotein SnC-SLO (ΔC101)-GAC^PR^. (**E**) SDS-PAGE analysis of purified SnT-Ferritin. (**F**) SDS-PAGE and Western blot analysis of covalent complex formation between glycoprotein and Fer nanoparticle. (**G**) Retention volume of nanovaccine in size-exclusion chromatography. (**H**) Representative TEM image of purified nanovaccine. (**I**) DLS analysis of nanovaccine and SnT-Fer.

Streptolysin O is a toxin protein widely secreted by GAS strains. Importantly, C-terminally truncated variants of SLO (SLO(ΔC101)) have been shown to elicit robust protective immune responses while completely abolishing hemolytic activity, thereby mitigating the safety concerns associated with the wild-type toxin [15]. Accordingly, this study aimed to develop a dual-antigen vaccine by co-utilizing GAC^PR^ polysaccharide and the SLO(ΔC101) protein. Conventional polysaccharide–protein conjugation strategies typically rely on random chemical cross-linking; however, recent studies have suggested that such approaches may compromise the immunogenicity of SLO, thereby diminishing overall vaccine efficacy [15,31]. To overcome this critical limitation, we employed PGCT to achieve site-specific conjugation between GAC^PR^ and the SLO(ΔC101) carrier protein. Subsequently, leveraging the highly specific SnoopCatcher/SnoopTag system, the resulting glycoconjugate was efficiently displayed on ferritin nanoparticles, ultimately yielding a polysaccharide–protein dual-antigen nanoparticle vaccine targeting GAS (Figure 1A).

To enable heterologous biosynthesis of GAC^PR^ in *E. coli*, the genes encoding *GacA–G* were amplified and cloned into the pBBR1MCS-2 vector to generate a polysaccharide expression plasmid. The sequence-verified construct was introduced into the *E. coli* chassis strain W3110 via electroporation. Following overnight cultivation, whole-cell lysates were analyzed by SDS–PAGE, and polysaccharide expression was confirmed by Western blotting using a commercial anti-GAC antibody. The presence of distinct polysaccharide bands in the transformants verified the successful heterologous expression of GAC^PR^ (Figure 1B). Given that the reducing terminus of GAC^PR^ is N-acetylglucosamine (GlcNAc), which is theoretically a cognate substrate for the oligosaccharyltransferase PglL, we next constructed the plasmid pET32a-PglL-SnC-SLO(ΔC101)-4573C. We genetically fused the functional glycosylation motif (CTB^4573^), which is exclusively recognized by the oligosaccharyltransferase PglL, to the C-terminus of the SLO(ΔC101) carrier protein. During co-expression, the catalytic activity of PglL directs the transfer of the GAC^PR^ polysaccharide chain strictly onto this engineered CTB^4573^ motif. This unique enzyme-substrate specificity naturally guarantees and demonstrates the site-specific nature of the glycosylation. This vector was designed to co-express PglL together with a fusion protein consisting of SnC, the antigen SLO(ΔC101), and the glycosylation acceptor sequon CTB^4573^. The PglL expression plasmid was co-transformed with the GAC^PR^ biosynthesis vector into *E. coli* W3110Δ*waaL*, followed by IPTG induction and overnight cultivation. Western blot analysis confirmed robust expression of the SnC-SLO(ΔC101) fusion protein and demonstrated efficient in vivo conjugation of GAC^PR^ to the acceptor protein mediated by PglL (Figure 1C). The glycoprotein-producing strain was subsequently scaled up to a 4 L culture, and the target glycoprotein was purified using a combination of affinity chromatography and SEC. Coomassie blue staining indicated that the final product was obtained at a purity exceeding 95% (Figure 1D). In parallel, SnT–ferritin nanoparticles were successfully expressed and purified via anion-exchange chromatography followed by SEC. Coomassie blue staining further confirmed the high purity of the purified SnT-ferritin product (Figure 1E).

The prepared polysaccharide–protein conjugate was mixed with ferritin nanoparticles in PBS and incubated at 4 °C overnight. SDS–PAGE analysis showed a marked increase in molecular weight following incubation, indicating effective conjugation between the polysaccharide–protein conjugate and ferritin nanoparticles. Western blot analysis further confirmed the successful assembly, as both anti-His and anti-GAC antibodies specifically recognized the polysaccharide–protein conjugate and the resulting nanoparticles, with coincident band positions (Figure 1F).

Comprehensive physicochemical characterization of the nanoconjugates was subsequently performed. SEC revealed that the nanoconjugate vaccine, exhibiting the highest molecular weight, eluted first, followed by ferritin nanoparticles and then the polysaccharide–protein conjugate, consistent with the expected size hierarchy (Figure 1G). The collected fractions from the distinct SEC peaks were further confirmed by SDS-PAGE, verifying that the earliest eluting peak corresponding to the largest hydrodynamic radius was the properly assembled nanovaccine. Negative-staining transmission electron microscopy demonstrated that the nanoparticles displayed a uniform and well-defined morphology (Figure 1H). Dynamic light scattering analysis showed an average particle diameter of 25.02 nm, which was substantially larger than that of the individual components prior to conjugation (Figure 1I). Collectively, these results confirm that the polysaccharide–protein conjugate was successfully anchored onto the surface of ferritin nanoparticles, validating the successful construction of the nanoconjugate vaccine.

### 3.2. The Dual-Antigen Nanoconjugate Induced Robust Immune Responses Against GAS

**Figure 2:**
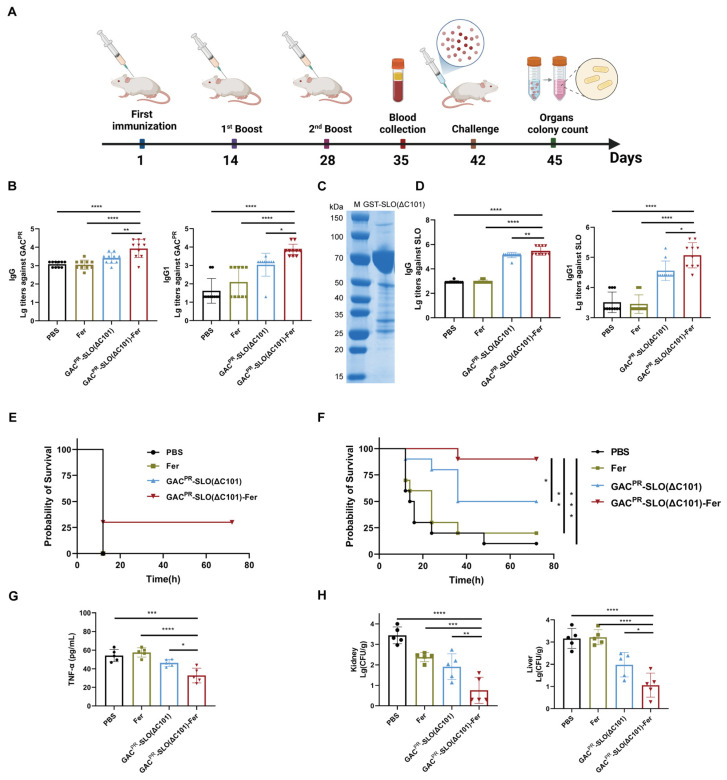
Protective efficacy of nanovaccine following active immunization. (**A**) Active immunization schedule in ICR mice. Created with BioRender.com. (**B**) Serum levels of GAC^PR^-specific IgG and IgG1 antibodies determined by ELISA (*n* = 10). (**C**) SDS-PAGE analysis of purified glycoprotein GST-SLO(ΔC101). (**D**) Serum levels of SLO-specific IgG and IgG1 antibodies determined by ELISA (*n* = 10). (**E**) Survival of mice after active immunization and GAS challenge with a high infection dose (8 × 10^8^ CFU per mouse). (**F**) Survival of mice after active immunization and GAS challenge with a reduced infection dose (5 × 10^8^ CFU per mouse). (**G**) Serum levels of TNF-α post-infection (*n* = 5). (**H**) Bacterial loads in liver and kidney 72 h after infection (*n* = 5). * *p* < 0.05, ** *p* < 0.01, *** *p* < 0.001, **** *p* < 0.0001. The data are presented as means ± SD.

We further evaluated the immunogenicity of the vaccine through a series of mouse immunization experiments. ICR mice were subcutaneously immunized with PBS, ferritin, GAC^PR^-SLO(ΔC101), or GAC^PR^-SLO(ΔC101)-Fer on days 0, 14, and 28, with PBS serving as the negative control (Figure 2A). Blood samples were collected from the tail vein on day 35, and serum levels of antigen-specific antibodies against SLO and GAC^PR^ were determined by ELISA. ELISA analysis showed that both the GAC^PR^-SLO(ΔC101) and GAC^PR^-SLO(ΔC101)-Fer groups elicited robust GAC^PR^-specific IgG responses, whereas the GAC^PR^-SLO(ΔC101)-Fer group induced significantly higher antibody titers, indicating enhanced humoral immunogenicity (Figure 2B). Further isotype analysis revealed a pronounced increase in GAC^PR^-specific IgG1 levels in mice immunized with GAC^PR^-SLO(ΔC101)-Fer, suggesting that nanoparticle-based antigen presentation effectively augmented humoral immune responses.

To exclude potential interference from the His tag, an additional ELISA was performed using GST-tagged SLO as the coating antigen (Figure 2C). Consistent with the GAC^PR^-specific antibody responses, mice immunized with GAC^PR^-SLO(ΔC101)-Fer exhibited significantly elevated IgG and IgG1 titers against SLO compared with control groups. Collectively, these results demonstrate that the GAC^PR^-SLO(ΔC101)-Fer nanovaccine efficiently induces strong and specific humoral immune responses against both the SLO protein antigen and the GAC^PR^ polysaccharide antigen (Figure 2D).

To evaluate the protective efficacy of the bioconjugate nanovaccine, immunized mice were challenged intraperitoneally with a lethal dose of GAS strain MGAS5005 (8 × 10^8^ CFU per mouse) on day 42, corresponding to 14 days after the final booster immunization. Mouse survival was monitored for 72 h post-challenge. In this initial experiment, all mice in the PBS, ferritin, and GAC^PR^-SLO(ΔC101) groups succumbed to infection within 16 h. In contrast, mice immunized with GAC^PR^-SLO(ΔC101)-Fer exhibited a survival rate of 30% (Figure 2E). To further validate the protective effect and optimize the challenge conditions, a second experiment was conducted using a reduced bacterial inoculum (5 × 10^8^ CFU per mouse). Under these conditions, the survival rate of the GAC^PR^-SLO(ΔC101)-Fer–immunized group increased markedly to 90%, which was significantly higher than that observed in all other groups (Figure 2F). Collectively, these results demonstrate that the GAC^PR^-SLO(ΔC101)-Fer nanovaccine confers robust protection against lethal challenge with the invasive M1 serotype GAS strain. Encouraged by the strong protection observed in lethal challenge experiments, we next assessed vaccine efficacy under nonlethal infection conditions that more closely mimic natural infection. Fourteen days after the third immunization, mice were intraperitoneally challenged with GAS MGAS5005 at a sublethal dose (1 × 10^8^ CFU per mouse). To evaluate systemic inflammatory responses, tail vein blood was collected on day 1 post-challenge, and serum levels of TNF-α were quantified by ELISA (Figure 2G). Mice immunized with GAC^PR^-SLO(ΔC101)-Fer exhibited significantly lower TNF-α levels compared with those in the PBS, Fer, and GAC^PR^-SLO(ΔC101) groups, which displayed pronounced inflammatory responses.

At day 3 post-infection, mice were humanely euthanized, and bacterial burdens in the liver and kidney were determined. The GAC^PR^-SLO(ΔC101)-Fer–immunized group showed the lowest bacterial loads in both organs, whereas substantially higher colony counts were detected in the control groups (Figure 2H). These findings indicate that immunization with GAC^PR^-SLO(ΔC101)-Fer markedly enhances bacterial clearance in vivo. Taken together, these results further confirm that the dual-antigen nanovaccine GAC^PR^-SLO(ΔC101)-Fer elicits strong protective immunity against GAS infection.

### 3.3. Passive Immunization Demonstrates Immediate Protective Efficacy of Nanovaccine-Induced Serum

**Figure 3:**
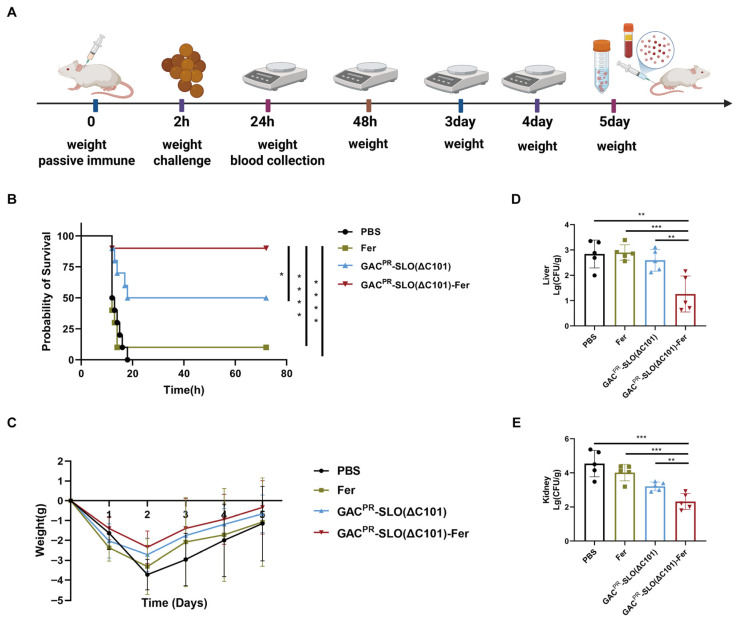
Passive immunization with nanovaccine immune serum against GAS infection. (**A**) Schematic of GAS challenge following passive immunization. Created with BioRender.com. (**B**) Survival rates after GAS challenge post-passive immunization. (**C**) Body weight changes after low-dose GAS infection. (**D**,**E**) Bacterial loads in liver (**D**) and kidney (**E**) at day 5 after infection (*n* = 5). * *p* < 0.05, ** *p* < 0.01, *** *p* < 0.001, **** *p* < 0.0001. The data are presented as means ± SD.

To further verify the protective efficacy of the conjugate nanovaccine and to assess its potential for rapid protection, we performed passive immunization experiments using serum collected from ICR mice immunized three times with PBS, Fer, GAC^PR^-SLO(ΔC101), or GAC^PR^-SLO(ΔC101)-Fer. Recipient mice were intraperitoneally administered 100 μL of five-fold diluted immune serum and challenged 2 h later with a median lethal dose of GAS MGAS5005 (3 × 10^8^ CFU per mouse) (Figure 3A). Following infection, mice began to succumb at approximately 12 h post-challenge, with the majority of deaths occurring between 12 and 20 h. Mice receiving serum from the PBS, Fer, or GAC^PR^-SLO(ΔC101) groups exhibited varying degrees of clinical symptoms, including lethargy, anorexia, adipsia, and tremors. In contrast, mice treated with serum derived from the GAC^PR^-SLO(ΔC101)-Fer–immunized group displayed markedly alleviated clinical manifestations. All mice in the PBS group succumbed to infection, whereas survival rates of 10% and 50% were observed in the Fer and GAC^PR^-SLO(ΔC101) groups, respectively. Notably, passive transfer of serum from GAC^PR^-SLO(ΔC101)-Fer–immunized mice conferred a survival rate of 90%, demonstrating superior protective efficacy (Figure 3B). To further evaluate protective effects under less stringent challenge conditions, the infectious dose of MGAS5005 was reduced to 1 × 10^8^ CFU per mouse, and body weight changes were monitored daily following infection. Mice receiving serum from the GAC^PR^-SLO(ΔC101)-Fer group exhibited the mildest body weight loss, indicating reduced disease severity (Figure 3C). At day 5 post-infection, mice were euthanized and bacterial loads in visceral organs were quantified. Consistent with survival and weight-loss data, significantly lower bacterial burdens were detected in the liver and kidney of mice treated with serum from the GAC^PR^-SLO(ΔC101)-Fer group compared with all other groups (Figure 3D,E). Collectively, these results demonstrate that passive transfer of nanovaccine-induced serum provides rapid and robust protection against GAS infection, effectively reducing disease severity, bacterial burden, and mortality. This finding highlights the strong protective capacity of vaccine-elicited humoral immunity and underscores the potential utility of the nanovaccine for emergency prophylaxis.

### 3.4. Safety Assessment of the Nanovaccine

**Figure 4:**
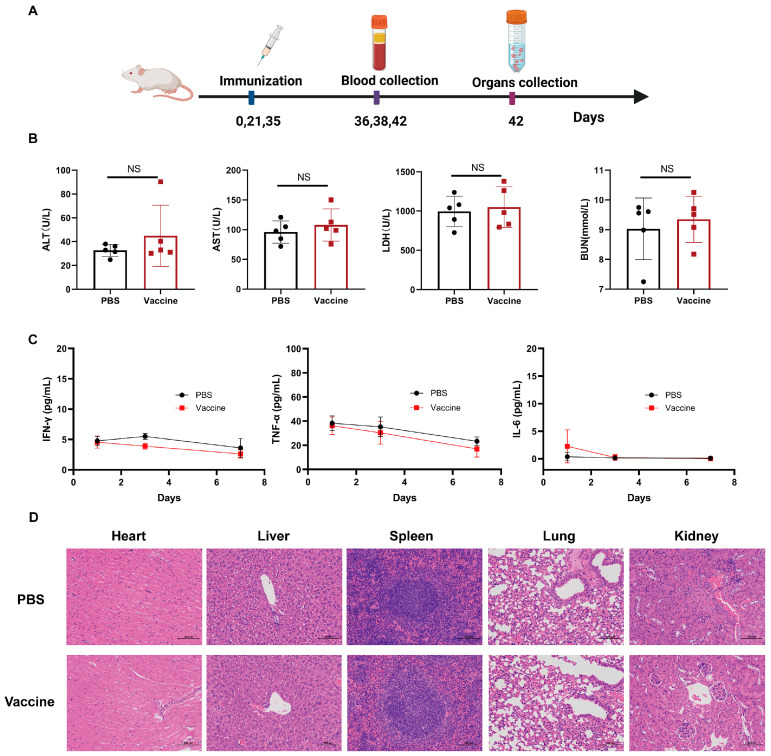
Safety evaluation of nanovaccine in mice. (**A**) Mice were subcutaneously injected with nanovaccine or PBS. A series of indicators were tracked at multiple time points. Created with BioRender.com. (**B**) Serum biochemical markers (ALT, AST, BUN, and LDH) on day 7 postinjection (*n* = 5). (**C**) Serum inflammatory factors (IFN-*γ*, TNF-*α*, IL-6) were detected at indicated time points (*n* = 5). (**D**) HE-stained tissue sections (heart, liver, spleen, lung, and kidney) were analyzed. The data are presented as means ± SD. NS, not significant.

The safety profile of the nanovaccine was systematically evaluated in vivo. Mice were subcutaneously immunized with GAC^PR^-SLO(ΔC101)-Fer (corresponding to 2.5 μg GAC^PR^), while control mice received PBS. Throughout the observation period, multiple safety-related parameters were assessed, including serum biochemical indices, inflammatory cytokines, and histopathological changes in major organs (Figure 4A). Serum biochemical analyses performed on day 7 post-immunization showed that alanine aminotransferase (ALT), aspartate aminotransferase (AST), blood urea nitrogen (BUN), and lactate dehydrogenase (LDH) levels in the vaccinated group remained within normal physiological ranges and were comparable to those in the PBS control group (Figure 4B). In parallel, serum levels of pro-inflammatory cytokines, including tumor necrosis factor-α (TNF-α), interferon-γ (IFN-γ), and interleukin-6 (IL-6), were measured. All cytokines were detected at very low levels, with no significant differences observed between the nanovaccine-treated and control mice, indicating the absence of excessive systemic inflammatory responses (Figure 4C). Furthermore, histopathological examination of the heart, liver, spleen, lung, and kidney by H&E staining revealed no observable pathological alterations or tissue damage in mice immunized with GAC^PR^-SLO(ΔC101)-Fer (Figure 4D). Collectively, these results demonstrate that the GAC^PR^-SLO(ΔC101)-Fer nanovaccine exhibits a favorable safety profile in mice, without inducing detectable systemic toxicity, inflammatory responses, or organ pathology.

## 4. Discussion

The substantial global health burden imposed by GAS—encompassing superficial infections, life-threatening invasive diseases, and post-infectious autoimmune sequelae such as rheumatic heart disease—underscores a critical and long-standing unmet need for an effective prophylactic vaccine [6]. Despite decades of intensive research, no licensed vaccine against GAS is currently available, reflecting both the biological complexity of this pathogen and the exceptionally stringent safety requirements imposed on vaccine candidates, particularly with respect to the risk of inducing autoimmune responses [32].

In this study, we report the rational design, successful construction, and comprehensive preclinical evaluation of a novel dual-antigen nanoconjugate vaccine, GAC^PR^-SLO(ΔC101)-Fer. By integrating multiple innovative strategies—including the use of an autoimmune-safe polysaccharide antigen, a conserved protein virulence factor, and a ferritin-based nanoparticle delivery system—this vaccine candidate effectively addresses several of the key obstacles that have historically hindered GAS vaccine development. Importantly, GAC^PR^-SLO(ΔC101)-Fer elicited robust antigen-specific humoral immune responses and conferred significant protection against lethal GAS challenge in murine models, highlighting its potential as a promising and safe next-generation GAS vaccine.

Antigen selection is a decisive determinant of both efficacy and safety in GAS vaccine development. The GAC is a highly conserved and indispensable surface structure shared across all GAS serotypes, rendering it an attractive universal vaccine target [33]. However, the native GAC contains a terminal N-acetylglucosamine (GlcNAc) side chain that exhibits molecular mimicry with human cardiac glycoproteins, raising a well-documented risk of autoimmune responses [14]. This concern has historically constituted a major barrier to the clinical translation of GAC-based vaccines. To circumvent this critical safety challenge, we adopted a heterologous biosynthesis strategy in *E. coli*, which intrinsically produces a modified GAC lacking the GlcNAc side chains, herein referred to as GAC^PR^. This approach preserves the conserved rhamnose backbone that harbors protective epitopes while theoretically eliminating the autoreactive determinants associated with molecular mimicry [34]. As such, the use of GAC^PR^ represents a cornerstone advance in reconciling broad serotype coverage with stringent safety requirements, substantially enhancing the translational potential of carbohydrate-based GAS vaccines. Despite these advantages, polysaccharide antigens such as GAC^PR^ are inherently T cell–independent, typically inducing weak and short-lived antibody responses with limited immunological memory, particularly in young children and other vulnerable populations. Conjugation to a protein carrier is therefore essential to convert the immune response into a T cell-dependent one. Our innovation lies not only in this conjugation strategy, but also in the deliberate choice of both the carrier antigen and the conjugation methodology. Rather than employing a heterologous carrier protein such as CRM197, we selected SLO, a highly immunogenic and broadly conserved GAS virulence factor [35,36]. SLO is secreted by nearly all clinical GAS isolates and is a well-established target of neutralizing antibodies that can inhibit cytolytic activity and enhance opsonophagocytic clearance [37]. To ensure safety while retaining immunogenicity, we utilized a truncated, non-hemolytic SLO mutant [36]. This design yields a rational dual-antigen vaccine that simultaneously targets a conserved polysaccharide and a functionally relevant protein antigen, thereby providing the potential for synergistic protective immunity against GAS infection.

The conjugation strategy itself is equally critical. Conventional polysaccharide–protein conjugation methods rely on random chemical cross-linking, which can obscure essential antigenic epitopes on both components and consequently compromise immunogenicity [31]. To circumvent this limitation, PGCT was employed in the present study, a strategy that enables site-specific, enzyme-mediated conjugation of GAC^PR^ to the SLO carrier protein in vivo. This precise, single-site attachment generates a homogeneous glycoconjugate while preserving the structural integrity and immunologically relevant epitopes of both the polysaccharide and the protein, thereby mitigating the epitope masking associated with random conjugation. Consistently, ELISA analysis demonstrated robust and concurrent antibody responses against both GAC^PR^ and SLO, validating the effectiveness of this site-directed conjugation strategy.

To further potentiate the immune response, we engineered a ferritin-based nanoparticle delivery system. Ferritin nanoparticles intrinsically self-assemble into uniform, highly ordered nanostructures that efficiently traffic to antigen-presenting cells and promote robust germinal center responses [38,39,40]. Leveraging the ultra-high-affinity SnC/SnT system, multiple GAC^PR^–SLO(ΔC101) glycoconjugates were covalently and site-specifically displayed on the surface of each ferritin nanoparticle, generating a high-density and repetitive antigen array [41]. Such multivalent antigen presentation is well established to enhance B cell receptor cross-linking and activation. In addition, the nanoscale dimensions of the particles facilitate efficient lymph node drainage and uptake by dendritic cells, while the SnC/SnT linkage ensures stable and oriented antigen presentation [41]. Consistent with these design principles, immunization with GAC^PR^–SLO(ΔC101)–Fer elicited significantly higher antigen-specific IgG and IgG1 titers than the unconjugated glycoconjugate mixture (GAC^PR^–SLO(ΔC101)). Importantly, this nanoparticle vaccine conferred a striking 90% survival rate following lethal challenge, substantially exceeding the protective efficacy of the soluble conjugate.

The breadth of protection conferred by the nanovaccine was further substantiated using multiple complementary metrics. In addition to preventing mortality, immunization significantly reduced bacterial burdens in major target organs and attenuated systemic inflammatory responses, as reflected by markedly lower post-challenge TNF-α levels. Passive immunization experiments provided compelling evidence that protection was predominantly antibody-mediated. Serum transferred from mice immunized with GAC^PR^–SLO(ΔC101)–Fer conferred rapid and robust protection to naive recipients, resulting in a 90% survival rate. This outcome closely correlated with the elevated antigen-specific antibody titers induced by the nanoparticle formulation, highlighting the central role of humoral immunity in mediating protection. Collectively, these findings underscore the potential of this vaccine platform not only for active immunization but also as a source for therapeutic antibody development or emergency prophylaxis.

Despite these encouraging results, several limitations of the present study should be acknowledged. First, protective efficacy was primarily evaluated against the M1 serotype, which is among the most prevalent invasive GAS strains but does not encompass the full antigenic diversity of circulating isolates. Future studies assessing efficacy across additional clinically relevant serotypes will be essential to establish the breadth of protection. Second, while our analyses focused on humoral immune responses and bacterial clearance, cellular immune responses were not examined. Given their importance in sustaining long-term immunity, further investigation into T cell-mediated responses will be necessary to fully characterize the durability and mechanistic basis of vaccine-induced protection [35].

## 5. Conclusions

This study presents a rationally engineered dual-antigen nanoparticle vaccine that addresses several longstanding challenges in the development of vaccines against GAS. We first achieved the heterologous biosynthesis of GAC^PR^ polysaccharide in *E. coli*, thereby eliminating the potential risk of autoimmune cross-reactivity associated with native GAC. To enhance immunogenicity while preserving key antigenic epitopes, we employed PGCT to enable site-directed conjugation of the GAC^PR^ to the SLO carrier protein. Furthermore, incorporation of ferritin nanoparticles as a delivery platform markedly improved antigen presentation and immune activation, resulting in substantially enhanced protective efficacy. Collectively, the integration of these rational design strategies yielded a vaccine candidate that demonstrates strong protective immunity, a favorable safety profile, and broad translational potential. Beyond validating this specific formulation, our findings highlight the power of combining precise conjugation chemistry with nanoscale antigen display to optimize conjugate vaccine performance. Importantly, this modular platform can be readily adapted to target other encapsulated bacterial pathogens through substitution of the polysaccharide and protein antigens, providing a scalable and versatile framework for next-generation bacterial vaccine development.

## Data Availability

The original contributions presented in the study are included in the article; further inquiries can be directed to the corresponding authors.

## Abbreviations

The following abbreviations are used in this manuscript:

GAS	Group A Streptococcus
PGCT	Protein Glycan Coupling Technology
GAC^PR^	Group A Carbohydrate polyrhamnose backbone
SnC	SnoopCatcher
SnT	SnoopTag
Fer	Ferritin
ARF	Acute rheumatic fever
RHD	Rheumatic heart disease
